# TOX3 is expressed in mammary ER^+^ epithelial cells and regulates ER target genes in luminal breast cancer

**DOI:** 10.1186/s12885-015-1018-2

**Published:** 2015-01-30

**Authors:** Akop Seksenyan, Asha Kadavallore, Ann E Walts, Brian de la Torre, Dror Berel, Samuel P Strom, Parinaz Aliahmad, Vincent A Funari, Jonathan Kaye

**Affiliations:** 1 Research Division of Immunology, Departments of Biomedical Sciences and Medicine, Cedars-Sinai Medical Center, 8700 Beverly Blvd., Davis 5089, Los Angeles, 90048 CA USA; 2 Department of Pathology and Laboratory Medicine, Cedars-Sinai Medical Center, Los Angeles, CA USA; 3 Samuel Oschin Comprehensive Cancer Institute, Cedars-Sinai Medical Center, Los Angeles, CA USA; 4 Center for Applied Molecular Medicine, University of Southern California, Keck School of Medicine, Los Angeles, CA USA; 5 Genomics Core Facility, Cedars-Sinai Medical Center, Los Angeles, CA USA; 6 Department of Pathology and Laboratory Medicine, University of California Los Angeles David Geffen School of Medicine, Los Angeles, CA USA; 7 Department of Medicine, David Geffen School of Medicine, University of California, Los Angeles, CA USA

**Keywords:** TOX3, Luminal B breast cancer, TFF1, HMG-box factor, ER target gene activation, Mammary epithelial progenitor

## Abstract

**Background:**

A breast cancer susceptibility locus has been mapped to the gene encoding TOX3. Little is known regarding the expression pattern or biological role of TOX3 in breast cancer or in the mammary gland. Here we analyzed TOX3 expression in murine and human mammary glands and in molecular subtypes of breast cancer, and assessed its ability to alter the biology of breast cancer cells.

**Methods:**

We used a cell sorting strategy, followed by quantitative real-time PCR, to study *TOX3* gene expression in the mouse mammary gland. To study the expression of this nuclear protein in human mammary glands and breast tumors, we generated a rabbit monoclonal antibody specific for human TOX3. *In vitro* studies were performed on MCF7, BT474 and MDA-MB-231 cell lines to study the effects of TOX3 modulation on gene expression in the context of breast cancer cells.

**Results:**

We found *TOX3* expression in estrogen receptor-positive mammary epithelial cells, including progenitor cells. A subset of breast tumors also highly expresses TOX3, with poor outcome associated with high expression of *TOX3* in luminal B breast cancers. We also demonstrate the ability of TOX3 to alter gene expression in MCF7 luminal breast cancer cells, including cancer relevant genes *TFF1* and *CXCR4*. Knockdown of TOX3 in a luminal B breast cancer cell line that highly expresses TOX3 is associated with slower growth. Surprisingly, TOX3 is also shown to regulate *TFF1* in an estrogen-independent and tamoxifen-insensitive manner.

**Conclusions:**

These results demonstrate that high expression of this protein likely plays a crucial role in breast cancer progression. This is in sharp contrast to previous studies that indicated breast cancer susceptibility is associated with lower expression of TOX3. Together, these results suggest two different roles for TOX3, one in the initiation of breast cancer, potentially related to expression of TOX3 in mammary epithelial cell progenitors, and another role for this nuclear protein in the progression of cancer. In addition, these results can begin to shed light on the reported association of TOX3 expression and breast cancer metastasis to the bone, and point to TOX3 as a novel regulator of estrogen receptor-mediated gene expression.

**Electronic supplementary material:**

The online version of this article (doi:10.1186/s12885-015-1018-2) contains supplementary material, which is available to authorized users.

## Background

Breast cancer is the second most common cancer in the US among women, accounting for approximately 40,000 deaths in 2013 [1]. The risk factors contributing to breast cancer initiation and progression include both environmental and genetic components, which interact in a complex and poorly understood fashion. In regard to the latter, genome wide association studies have been performed to identify novel disease risk alleles. This approach has identified high frequency and low penetrance disease-associated single nucleotide polymorphisms (SNP) in a number of genes, including that encoding the HMG-box nuclear protein TOX high mobility group box family member 3 (TOX3) [2,3]. Despite this association, little is known concerning the expression pattern or biological functions of TOX3 in breast cancer or in mammary epithelial cells.

The HMG-box superfamily is defined by a ~80 amino acid DNA-binding domain, and individual members function in regulation of gene expression, chromatin remodeling, genomic stability, DNA repair, and other DNA-dependent cellular processes (reviewed in ref. [4]). The thymocyte selection-associated HMG box protein (TOX) subfamily of HMG-box proteins contains four evolutionarily conserved proteins: TOX, TOX2, TOX3, and TOX4. TOX family members share a near identical DNA-binding domain and are thought to interact with DNA in a structure-dependent but sequence-independent fashion [5]. While the N-terminal domains of these proteins share some similarity and have transactivation activity ([5], and J. Kaye data not shown) the C-terminal domains are distinct, suggesting the possibility of non-overlapping functions. The founding member of this protein family, TOX, plays a key role in the development of multiple aspects of the immune system [6-8] while the *in vivo* function of TOX3 remains to be identified.

*TOX3* risk-allele carriers have been reported to develop more lobular breast tumors, and patients with this SNP who develop luminal A (LumA) breast tumors have shorter overall survival [9]. Rare allele homozygotes were also found to have a higher risk for distant metasteses [10], although molecular subtype of the resulting tumors is uncertain. Recently, Lupien and colleagues [11] used a bioinformatics approach to identify SNPs directly implicated in increased breast cancer risk. The *TOX3* SNP causative of increased cancer risk is located 18 kb upstream of the *TOX3* transcription start site. This SNP alters a FOXA1 binding site, with disease susceptibility associated with enhanced FOXA1 binding, disrupted enhancer function, and a decrease in *TOX3* gene expression [11]. This was consistent with earlier work where a linked disease-associated SNP was correlated with lower *TOX3* mRNA in breast cancers [9,12]. The inverse association between TOX3 expression and disease risk has led to the suggestion that TOX3 may act as a tumor suppressor [11]. In addition, rare mutations of TOX3 in breast tumors have been reported [13]. However, some *TOX3* expressing tumors are associated with adverse outcome [9], and increased expression of *TOX3* mRNA has been implicated in breast cancer metastatic to bone [14]. Thus, whether TOX3 plays dual and opposing roles in cancer initiation and progression remains to be determined.

Here we show that *TOX3* is specifically expressed in the estrogen receptor alpha positive (ER^+^) subset of murine mammary luminal epithelial cells, including a recently identified progenitor cell subset. Using a novel anti-TOX3 monoclonal antibody developed by our laboratory, we confirmed high expression of TOX3 in human breast tissue samples enriched for ER^+^, progesterone receptor positive (PR^+^), and FOXA1^+^ luminal epithelial cells. The TOX3 protein was also highly expressed in a subset of breast cancers, predominantly among histologically defined luminal B (LumB) and LumBHer2^+^ breast cancer. Since *TOX3* overexpression is associated with poorer outcome in patients with LumB cancer, we also sought to identify genes whose expression would be influenced by expression of this nuclear protein. In the MCF-7 breast cancer cell line, TOX3 upregulates a subset of ER target genes in addition to genes involved in cell cycle, cancer progression and metastasis. The former includes *TFF1*, which was upregulated by TOX3 even in the absence of estrogen. In addition, TOX3 induces enhancer RNAs that have been implicated in TFF1 gene regulation. Conversely, loss of TOX3 from LumB BT474 breast cancer cells led to decreased proliferation. Together, this work implicates a role for TOX3 in tumor progression/metastasis as well as modulation of ER-dependent responses, potentially explaining the poorer outcome of the *TOX3*-high subset of LumB breast cancers. The apparent paradox whereby low *TOX3* is associated with cancer risk and high expression is associated with poor outcome is discussed in relation to *TOX3* expression in a subset of normal mammary epithelial cells.

## Methods

### Mice

All mice were bred at the Cedars-Sinai Medical Center and kept under specific pathogen free conditions, or purchased from the Jackson Laboratory (Bar Harbor, ME, USA). The CSMC Institutional Animal Care and Use Committee approved use of animals (IACUC#3376).

### Cell culture and transfection

MCF-7, BT474, and MDA-MB-231 cells were generously provided by Dr. H. Phillip Koeffler (Cedars-Sinai). HEK293T cells were provided by Dr. D. Nemazee (The Scripps Research Institute). Cells were maintained in DMEM (Life Technologies, Carlsbad, CA, USA) containing 10% fetal bovine serum (FBS) (Atlanta Biologicals, Flowery Branch, GA, USA). For experiments involving estrogen depletion, media was replaced by phenol-free DMEM (Life Technologies) containing 5% charcoal/dextran-treated FBS (Atlanta Biologicals).

X-tremeGENE (Roche, Indianapolis, IN, USA) was used for the transfection of plasmids and Lipofectamine 2000 (Life Technologies) for transfection of siRNAs into MCF-7 and HEK293T cells. Lipofectamine 2000 was used for transfection of MDA-MB-231 cells. Two validated *TOX3* or *ER* Stealth RNAi duplexes and Stealth RNAi negative control duplexes (Life Technologies) were tested. Depletion of mRNA expression was measured by quantitative real-time PCR (qRT-PCR), and the most efficient RNAi duplex was identified for further use. RNAi or negative control transfected BT474 cells were also processed for protein depletion as assessed by Western blot. The human *ESR1* expression plasmid was purchased from Addgene (Cambridge, MA, USA).

In some experiments, MCF-7 cells were transfected and after 8 hours switched to estrogen-depleted conditions. After an addiitonal 40 hours of cullture, cells were assessed for gene expression by qRT-PCR, or GFP^+^ cells were sorted for microarray analysis.

The gene encoding the long form of TOX3 was cloned from a breast cancer sample, sequenced, and compared with publicly available sequences to rule out mutation. For stable transfection, MCF-7 cells were transfected with an IRES-GFP containing empty expression vector (V) or human TOX3 encoding vector (T). Stable transfectants were selected in 2 mg/ml active G418. Upon growth, cells expressing equivalent levels of GFP were isolated from each line by cell sorting. Expression of *TOX3* was confirmed by qRT-PCR and Western blot.

MDA-MB-231 cells were cultured under estrogen-depleted conditions for 24 hours before transfection. Gene expression was measured 48 hours after transfection.

### Generation and validation of anti-TOX3 monoclonal antibody

Anti-TOX3 monoclonal antibody was produced in conjunction with Epitomics (Burlingame, CA, USA). Rabbits were immunized with a mixture of N- or C-terminal peptides derived from the long form of TOX3. After multiple boosting with the peptide mixture, serum was isolated for analysis. To screen for production of specific antibodies against the native protein, protein lysates from HEK293T cells transfected either with empty vector or a TOX3-encoding expression plasmid were analyzed by Western dot blot using immune sera. Hybridomas were then produced from an animal with high titer. The resulting antibody-containing culture supernatants were screened for specific reactivity against *TOX3* transfected HEK293T cells. Based on this analysis, one clone specific for the N-terminal TOX3 peptide was chosen for detailed analysis. Anti-TOX3 antibody, henceforth referred to as AJ-33, was purified by protein A affinity chromatography and was validated in multiple assays reported here.

### Mammary cell isolation and flow cytometry

All reagents were from StemCell Technologies (Vancouver, BC, Canada) unless otherwise specified. Mammary glands from 8–20 week-old virgin female C57Bl/6 mice, were digested for >8 h at 37°C in EpiCult-B with 5% FBS, 300U collagenase and 100U hyaluronidase. After vortexing and lysis of the red blood cells in NH_4_Cl, a single cell suspension was obtained by sequential dissociation of the fragments by gentle pipetting for 1–2 minutes in 0.25% trypsin, and then 2 minutes in 5 mg/ml dispase II plus DNase I followed by filtration through a 40-mm mesh.

Antibodies were obtained from eBioscience/affymetrix (San Diego, CA, USA). Mouse mammary cells were preblocked with anti-CD16/CD32 and then incubated with the following primary antibodies CD31-biotin, CD45-biotin, Ter119-biotin, BP-1-biotin, EpCAM-AF647, CD49f-AF488, or CD49f-Pacific Blue, CD49b-PE and Sca1-PE/Cy7. CD45, Ter119, CD31 and BP-1 were used to identify contaminating haematopoietic cells, endothelial cells and a proportion of stromal cells, respectively (collectively termed Lin^+^ cells). Biotin-conjugated antibodies were detected with streptavidin-APC/Cy7. Cells were analysed using an LSRII and specific cell populations isolated using a FACSAria III (BD Biosciences, San Jose, CA, USA). Flow cytometry data were analysed using FlowJo™ software (Tree Star, Ashland, OR, USA).

For surface CXCR4 detection, 5 × 10^5^ cells were incubated at 4°C for 45 min with 5 μg/ml of the specific monoclonal antibody to CXCR4 conjugated to PE. Cells were washed twice with PBS and resuspended in 500 μL of PBS for analysis.

### Boyden chamber migration assay

For migration studies, cells were first grown in phenol red- and serum-free DMEM for 24–48 hours. Subsequently, 2 × 10^5^ cells were seeded in 500 μl serum-free DMEM in the upper chamber of a 24 well transwell system (ThermoFisher Scientific, Waltham, MA, USA). Phenol red-free DMEM supplemented with 10% charcoal stripped dextran treated FBS was used as a chemoattractant in the lower wells. Phenol red- and serum-free DMEM was used as a negative control to assess basal migration rates. After 24 hours, membranes were scrubbed to remove nonmigrated cells and membranes were removed and stained using Diffquik (ThermoFisher Scientific). Migrated cells were visualized by microscopy and the number of cells in the center of each well was counted. Data are represented as number of migrated cells per field of view ± SD for triplicate samples.

### Cell proliferation

Cells were seeded in 24-well plates (1 × 10^4^ cells per well) and cultured for 10 days (MCF-7) or 14 days (BT474) in appropriate culture medium. Numbers of viable cells were determined using Trypan Blue (Sigma-Aldrich, St. Louis, MO, USA).

### qRT-PCR

Primary breast cancer RNA samples were purchased from BioServe (Beltsville, MD, USA). Otherwise, RNA extraction was performed using the RNAeasy kit followed by cDNA production using the Quantitect Reverse Transcription kit (Qiagen, Valencia, CA, USA). qRT-PCR was performed using SYBR Green PCR Master Mix and commercially available primers (Qiagen) were used unless indicated. *GAPDH*, *MRPL*, or *ACTB* expression served as housekeeping gene controls. Relative gene expression was analyzed using the 2-ΔΔCt method. Primer sequences to detect *TOX3* variants are available upon request. For pre-mRNA measurements, RNA was isolated and qRT-PCR performed as above, but using primers that specifically detect pre-spliced *TFF1* mRNA as previously described [15].

### Nuclear extractions and Western blot

Nuclear protein was isolated using a Nuclear Extract Kit (Active Motif, Carlsbad, CA, USA). 20 μg of protein lysate was subjected to Western blotting using AJ-33 or anti-Actin (EMD Millipore, Billerica, MA, USA) antibodies as detected by peroxidase-conjugated anti-rabbit IgG antibody (Bio-Rad, Hercules, CA, USA) and chemiluminescence.

### Immunofluorescence

HEK293T cells were grown on eight-well chamber slides. After 48 hours of transfection, cells were fixed in 4% paraformaldehyde, blocked in 10% normal goat serum for 1 hour, and stained with primary antibodies overnight at 4°C. Goat anti-rabbit antibody conjugated to PE (Jackson ImmunoResearch Laboratories, West Grove, PA, USA) was used to detect primary antibodies. Slides were mounted with 4',6-diamidino-2-phenylindole (DAPI) (Life Technologies) to visualize nuclei.

### Tissue samples and immunohistochemistry

Immunohistochemical detection of TOX3 was performed on 4-μm sections of formalin fixed paraffin embedded tissue stained with AJ-33. Staining was done using an automated slide stainer (Dako, Carpinteria, California, USA). Antigen retrieval was performed using the Dako PT Link Module and low pH buffer. Staining was visualized using the Dako Envision + Rabbit Detection System. Slides were subsequently counterstained with Mayer’s hematoxylin (Sigma-Aldrich). Three commercially available tissue arrays (Pantomics, Richmond, CA, USA) that contained a total of 210 primary breast cancer samples in duplicate were analyzed for TOX3 expression. Some samples were destroyed during the processing and therefore removed from analysis. Thus, the TOX3 histological data shown here are derived from a total of 188 breast tumors. Other histological data that were associated with individual tumors on the tissue array were supplied by the manufacturer. For some histological analysis, the Department of Pathology and Laboratory Medicine at CSMC supplied normal breast tissue obtained following mammoplasty. Samples were obtained under a waiver of consent and provided in an anonymous fashion, so that the connection to individual patients was destroyed prior to their analysis. This work was performed under Cedars-Sinai Medical Center’s Institutional Review Board Study Number: Pro 00033387.

### Molecular subtyping and microarray analysis

Publicly available (GSE12093, GSE11121, GSE7390, GSE2034) breast cancer microarray expression data was compiled and normalized using 820 patient samples run only on Affymetrix (Santa Clara, CA, USA) gene chips. Samples were molecularly subtyped using the CIT classification algorithm R package provided by the authors [16]. Multiple probes of the *TOX3* gene were averaged. A box-and-whisker plot was generated demonstrating the levels of *TOX3* mRNA expression within each molecular subtype.

Human gene expression analysis was performed using Affymetrix microarrays. In brief, RNA was isolated using the RNAeasy kit (Qiagen), and RNA quality was assessed using a Nanodrop (Thermo Scientific, Wilmington, DE, USA) spectrometer and an Agilent 2100 Bioanalyzer. RNA was reverse transcribed and hybridized to HuGene 1.0 Affymetrix arrays and processed according to manufacturer’s recommendations. Data were normalized using Justplier algorithm by Affymetrix available in Bioconductor v2.0 and R v.3.0. Since microarray signals are not quantitative at lower and higher values, signal thresholds for floor and ceiling were set for all samples. Two-way unsupervised hierarchical clustering was performed to assess unbiased gene expression patterns associated with samples. Genes whose intensity varied at least two fold between any two arrays independent of treatment were analyzed. Dendrograms were then constructed from a distance matrix containing Pearson correlations calculated iteratively between the four samples and 223 genes.

### Statistical analyses

Two-tailed Student *t* tests were used for significance testing; *P* < 0.05 was considered significant with * = *P* <0.05, ** = *P* <0.01, and *** = *P* <0.001. Error bars represent standard deviation of replicates.

### Availability of supporting data

Raw binary CEL files as well as normalized data for microarray analysis were deposited in Gene Expression Omnibus at NCBI (http://www.ncbi.nlm.nih.gov/geo/) and can be obtained using Accession # GSE57856.

## Results

### TOX3 expression in mouse and human breast epithelium

**Figure 1:**
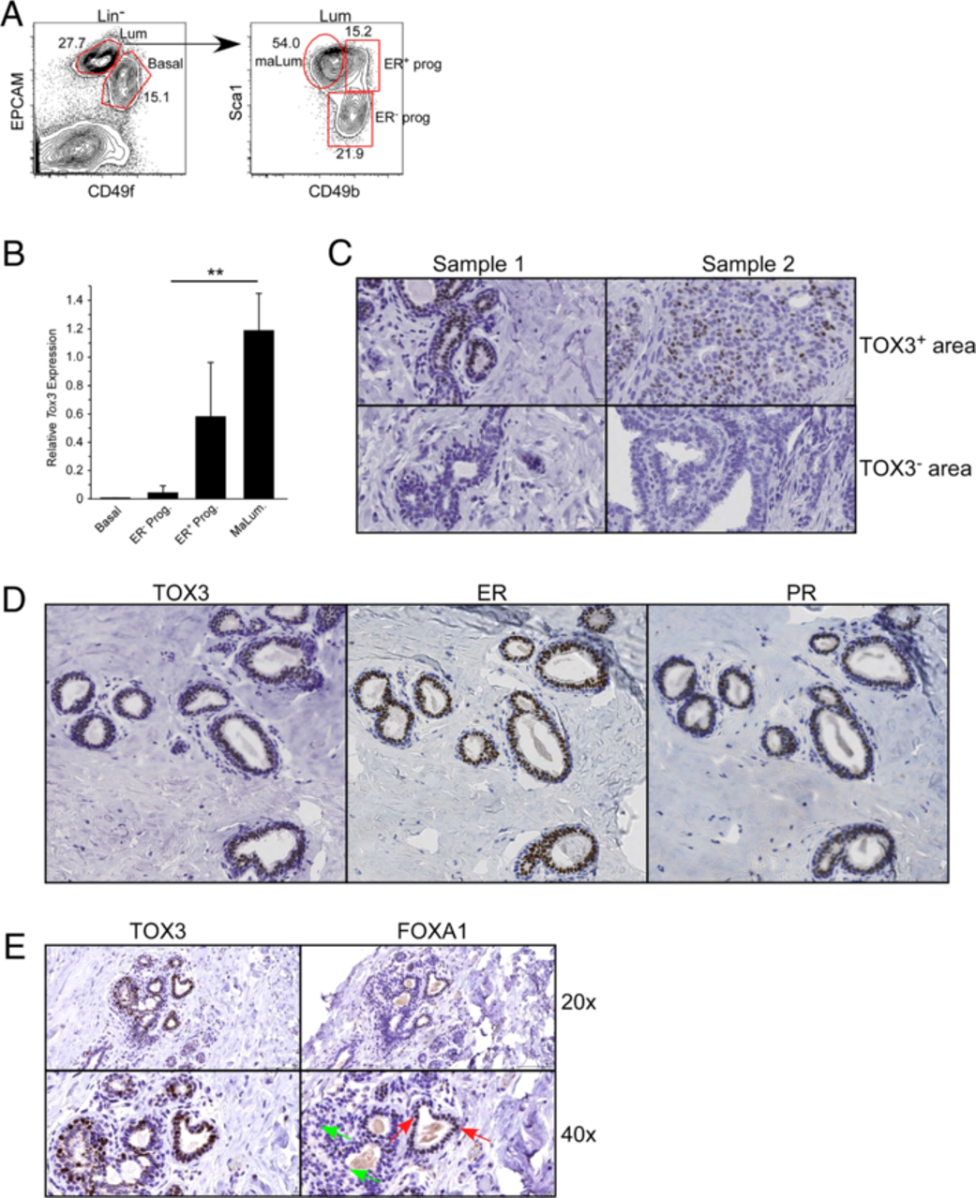
**TOX3 is expressed in mouse and human mammary epithelium. A**. Gating strategy used to sort mouse mammary epithelial cell populations is shown. Contour plot on the right is gated on the luminal epithelial population as shown. **B**. qRT-PCR for mouse *Tox3* in the indicated sorted cell populations. Data are expressed as mean ± SD of three independent sorts. **C**. Immunohistochemical staining of TOX3 in reduction mammoplasty samples showing areas of high and low TOX3 protein expression within an individual (magnification, 40X). **D**. Immunohistochemical staining of serial sections with antibodies specific for TOX3, ER, and PR (magnification 20X). **E**. Immunohistochemical staining of serial sections for TOX3 and FOXA1 at indicated magnification. Examples of FOXA1^+^ and FOXA1^−^ nuclei are indicated by red and green arrows, respectively.

TOX3 expression has not been well defined in normal mammary epithelial cells. Indeed, TOX3 was reported to be poorly expressed in total breast tissue [17]. We utilized previously identified subsets of murine mammary epithelial cells [18] to analyze *Tox3* expression in isolated basal epithelial cells (CD49f^+^EpCAM^low^), ER^−^ luminal progenitor cells (CD49f^+^EPCAM^low^CD49b^+^Sca1^−^), ER^+^ luminal progenitor cells (CD49f^+^EpCAM^low^CD49b^+^Sca1^+^), and ER^+^ mature luminal cells (CD49f^+^EPCAM^low^CD49b^−^Sca1^+^) (Figure 1A). *Tox3* was not detectable in basal cells, and minimally expressed in ER^−^ luminal progenitor cells (Figure 1B). In contrast, there was higher expression of *Tox3* mRNA in ER^+^ progenitors and ER^+^ mature luminal cells (Figure 1B). The transcriptome of human counterparts of these cell populations has recently been reported [18]. Analysis of this expression data demonstrated a similar pattern of *TOX3* expression, with highest levels of *TOX3* in ER^+^ luminal progenitors and ER^+^ mature luminal cells (Additional file 1: Figure S1).

To further characterize TOX3 expression in human mammary epithelium at the protein level, we produced a rabbit anti-human TOX3 monoclonal antibody (AJ-33). To test for specificity, we transfected HEK293T cells with a CMV promoter-based vector expressing TOX3 and GFP, the latter under control of an IRES. Transfected cells were mixed at various ratios with non-transfected cells and a Western blot was performed using AJ-33 (Additional file 2: Figure S2A). A single band of apparent molecular weight (MW) of ~65 kDa was detected in a quantitative manner only in transfected cells, correlating with qRT-PCR results (Additional file 2: Figure S2A). To determine subcellular localization of TOX3, we performed confocal microscopy on TOX3 transfected cells, using empty vector (GFP alone) transfected cells as a control. TOX3, like TOX [5], contains a putative bipartite nuclear localization signal adjacent to the HMG-box. Consistent with this, TOX3 expression as determined by AJ-33 staining was localized to the nucleus in transfected cells (Additional file 2: Figure S2B). This antibody was then used to study protein expression of TOX3 in human mammary epithelium.

Formalin-fixed paraffin-embedded sections of ten mammoplasty breast samples were immunostained with AJ-33. Variable TOX3 expression was observed in mammary epithelium, with some areas exhibiting no TOX3^+^ cells and in other areas showing a high proportion of TOX3^+^ cells (Figure 1C). As in transfected cells, TOX3 staining was confined to the cell nucleus. TOX3 protein was detected solely in luminal epithelial cells; no staining was observed in stromal or myoepithelial cells, consistent with the absence of *TOX3* gene expression in murine and human basal epithelial cell populations noted above.

As *TOX3* mRNA was primarily detected in ER^+^ luminal epithelial cells, we asked whether TOX3 protein expression was associated with this cell subset in human mammary gland tissue. Serial sections demonstrated that areas of mammary tissue with a high proportion of TOX3^hi^ cells also expressed ER, the ER target progesterone receptor (PR), and the ER pioneer factor FOXA1 (Figure 1D,E). Interestingly, these same areas had a very high proportion of ER^+^ luminal cells. However, not all areas with a high proportion of ER^+^ luminal cells were TOX3^+^ (data not shown).

### *TOX3* is expressed in a subset of breast cancer and correlates with poor outcome

**Figure 2:**
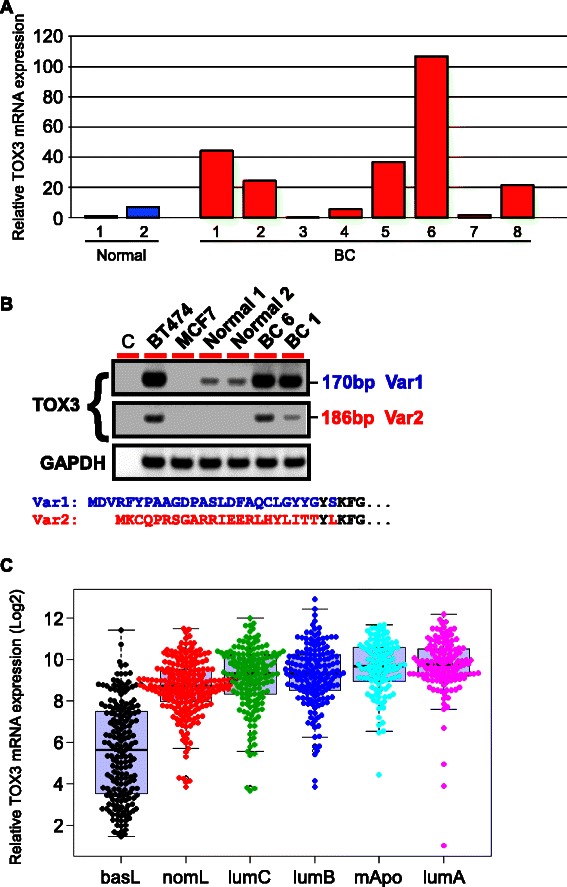
***TOX3*mRNA is expressed in multiple molecular subtypes of breast cancer. A**. Expression of *TOX3* was determined by qRT-PCR in breast cancer (red) and normal breast (blue) samples, relative to housekeeping gene *MRPL*. Relative expression was then normalized to ‘Normal 1’. **B**. RT-PCR analysis of the long (Var1) and short (Var2) variants of *TOX3*, and *GAPDH*, in breast cancer cell lines (BT474, MCF-7), two normal breast samples, and two primary breast cancer samples BC6 and BC1, as in **(A)**. **C**. Relative *TOX3* mRNA expression in six intrinsic molecular subtypes of breast cancer.

Analysis of a series of samples from primary breast cancer revealed extreme variability in *TOX3* mRNA expression ranging from undetectable to 100-fold over that detected in two normal breast tissue samples (Figure 2A). Two *TOX3* splice variants predicted to encode proteins with distinct N-terminal sequences (NP_001073899.2 and NP_001139660.1) have also been reported (Figure 2B). However, nothing is known about the expression of these isoforms in normal tissue or breast cancer. Using a common downstream primer and specific upstream primers that allowed us to distinguish these variants by PCR, the long form of *TOX3* appears as the predominant mRNA in breast cancer cell lines and two primary breast tumors (Figure 2B). As expected, lower levels of *TOX3* were found in total normal breast tissue, with only the long form detected in these samples (Figure 2B).

Breast cancer is a heterogeneous disease and various attempts have been made to define molecular subtypes with prognostic value. To determine if the expression of *TOX3* was associated with a particular molecular subtype of breast cancer, we analyzed a publicly available microarray data set of primary human breast cancers. Tumors were classified into six intrinsic molecular subtypes, LumA, LumB, luminal C, basal, normal-like and molecular apocrine, as previously reported [16]. *TOX3* was expressed across multiple molecular subtypes, including luminal, molecular apocrine, and normal-like (Figure 2C). In contrast, basal subtype cancers rarely expressed *TOX3* (Figure 2C), consistent with a previous report [19].

**Figure 3:**
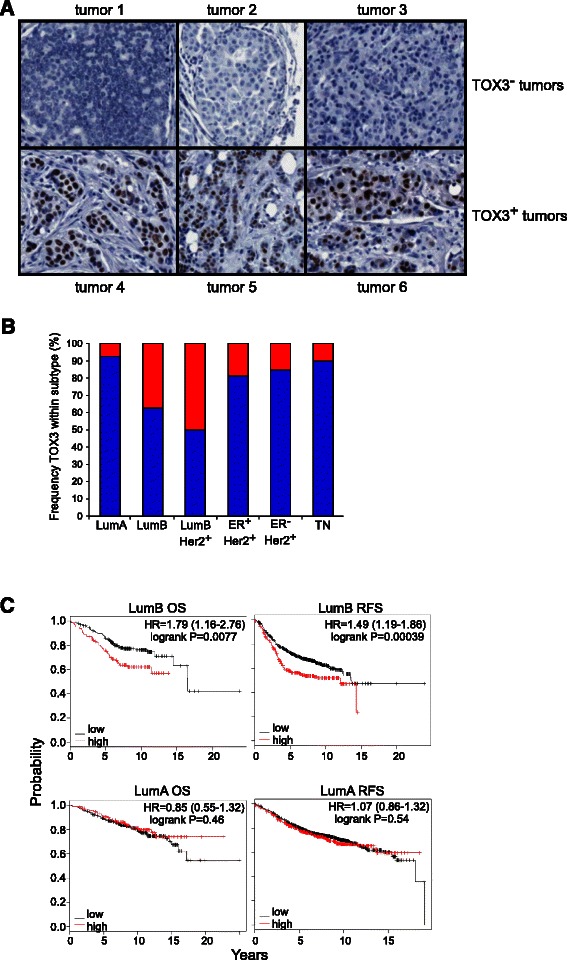
**TOX3 is highly expressed in a subset of LumB tumors and is associated with poor prognosis. A**. Examples of TOX3 protein expression in six primary breast cancer samples. **B**. Proportion of TOX3^hi^ (red) tumors among histologically defined breast cancer subtypes on tissue array of 188 breast tumors. **C**. Kaplan-Meier plots showing overall survival (OS) and recurrence free survival (RFS) of patients bearing *TOX3*^hi^ tumors of the LumB and LumA subtypes. Hazard ratios, confidence intervals, and logrank P values are shown.

To assess expression of the TOX3 protein in breast tumors, we stained a tissue array with AJ-33 antibody. TOX3 protein expression ranged from undetectable to highly expressed, both in frequency of cells stained and intensity of staining (Figure 3A and data not shown), mirroring the high variability in *TOX3* mRNA found in tumor samples (Figure 2A). TOX3 expression was localized to the tumor cell nucleus, and was not detected in surrounding stroma. Using a threshold of >10% of stained epithelial cells, ~15% of the cancers expressed TOX3. We further classified tumors on the array as LumA (ER^+^Her2^−^, Ki67 < 14%), LumB (ER^+^Her2^−^, Ki67 ≥ 14%), LumBHer2, (ER^+^Her2^+^, Ki67 ≥ 14%), Her2 (ER^−^Her2^+^), and triple negative (ER^−^PR^−^Her2^−^), based on accompanying histological data. In contrast to the microarray data, LumB and LumBHer2 tumors showed clear enrichment for TOX3 expression compared to other subtypes (Figure 3B). This may reflect differences in molecular compared to histological subtyping, discordance between mRNA and protein expression (although we have not observed this in cell lines, data not shown), or the quantitative limitations of array-based transcriptome analysis, emphasizing the importance of protein expression analysis. Since a substantial subset of LumB breast tumors highly expressed TOX3, we sought to correlate *TOX3* expression with outcome, using the on-line KM-Plotter for breast cancer [20]. Patients with LumB tumors were split by the upper tertile of TOX3 expression. High expression of TOX3 mRNA expression was associated with a highly significant decrease in overall and recurrence free survival (Figure 3C). Most interestingly, no such association was seen among patients with LumA tumors (Figure 3C).

### TOX3 regulates genes involved in breast cancer aggressiveness in MCF-7 cells

**Figure 4:**
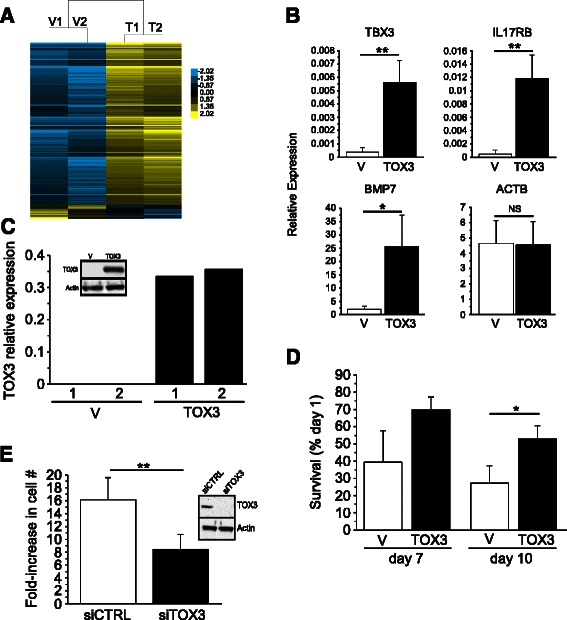
**Expression of TOX3 in MCF-7 cells leads to upregulation of genes implicated in cancer progression. A**. Two-way hierarchical clustering of gene expression changes in MCF-7 cells upon transient expression of *TOX3*. **B**. qRT-PCR validation of selected genes from microarray result. Data are expressed as mean ± SD of three independent experiments. **C**. *TOX3* mRNA and protein expression (inset) in MCF-7 stable cell lines. **D**. Cell survival of stably transfected MCF-7 cell lines at indicated days, grown in estrogen-depleted medium. Data are expressed as mean ± SD of three cultures. **E**. BT474 cell growth upon siRNA-mediated knockdown of TOX3. Data are expressed as mean ± SD of five independent experiments.

MCF-7 is an ER^+^ luminal type breast cancer cell line with low TOX3 expression (Figure 2B). To determine the effects of TOX3 on gene expression, we performed transient transfection of these cells with an expression vector encoding the predominant form of TOX3 in conjunction with GFP or, as a control, GFP alone. Vector control or *TOX3*-transfected cells expressing equivalent levels of GFP were isolated by cell sorting and subjected to whole transcriptome microarray analysis. As previous data suggested that TOX3 could regulate gene expression from promoters carrying an estrogen response element [17], these experiments were carried out under estrogen-depleted conditions. Unsupervised hierarchical clustering of independent biological replicates suggests that TOX3-expressing MCF-7 had coordinated gene expression changes that were distinct from vector controls (Figure 4A, Additional file 3: Figure S3). In this context, TOX3 acted primarily as a transcriptional activator, with expression of the protein upregulating a large number of genes and downregulating a much smaller subset of genes, although we can not distinguish direct from indirect gene targets (Figure 4A).

The ability of TOX3 to upregulate a number of genes implicated in breast cancer or mammary gland development, including TBX3 [21], BMP7 [22,23], and IL17RB [24], was confirmed by qRT-PCR in transiently transfected cells under estrogen-depleted conditions (Figure 4B). We also subjected these microarray data to Ingenuity pathway analysis (Additional file 4: Figure S4). Upregulation of multiple members of gene networks involved in cell cycle, DNA repair, and cancer was observed, including upregulation of BRCA1 and 2, and BIRC5 (Survivin) genes.

To study the biological consequences of TOX3 over expression, two independent MCF-7 cell lines were generated and mRNA and protein analyses confirmed expression of TOX3 (Figure 4C). Given that MCF-7 cells are highly dependent on estrogen for growth and survival and that TOX3 could upregulate genes important for cell cycle, we estrogen-depleted the stably transfected cells and monitored their growth. Although TOX3 did not promote cell proliferation in the absence of estrogen, we observed significantly more cells in TOX3 expressing cell cultures at day 10 relative to vector control cells, suggesting some resistance to estrogen depletion (Figure 4D). These results suggested that TOX3 might play a role in enhancing tumor cell proliferation and/or survival. To address this, we performed siRNA-mediated knockdown of TOX3 in BT474 cells. BT474 is a LumB cell line [25] that highly expresses TOX3 (Figure 2B), consistent with expression of TOX3 protein in a significant proportion of primary LumB tumors as described above. Decrease in TOX3 expression significantly inhibited BT474 expansion over a 12-day period (Figure 4E).

### TOX3 promotes migration of MCF-7 cells and is upregulated by IGF-1

**Figure 5:**
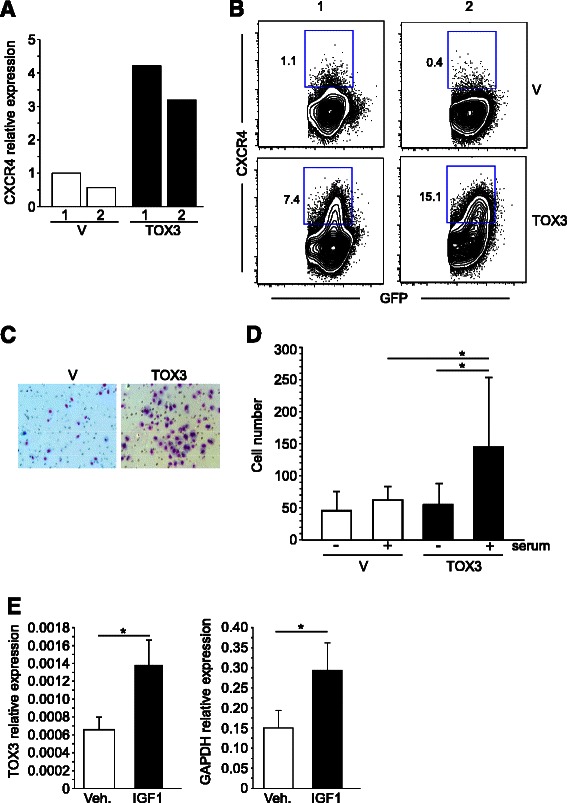
**TOX3 enhances MCF-7 migration and is upregulated by IGF-1. A**. qRT-PCR and **B**. FACS analysis, for CXCR4 expression in two independently generated vector or *TOX3* stably transfected MCF-7 cell lines. **C**. Representative images of migratory cells in vector control (V) or TOX3-expressing (TOX3) cell lines. **D**. Quantification of migratory cells in vector and TOX3 transfected cell lines using FBS as chemo-attractant. Data are expressed as mean ± SD of three independent experiments. **E**. qRT-PCR analysis of *TOX3* and *GAPDH* (a positive control for IGF-1 response), relative to *ACTB*, in MCF-7 cells treated with IGF-1. Data are expressed as mean ± SD of three independent experiments.

Among the genes upregulated by TOX3 in our microarray analysis (Figure 4a) was the well-studied pro-metastatic gene *CXCR4* [26-28]. To determine if cells stably expressing TOX3 also maintained expression of this chemokine receptor at the level of RNA and protein, we performed qRT-PCR and FACS analysis, respectively. Both analyses demonstrated upregulation of CXCR4 in cells that expressed TOX3 as compared to vector control cell lines (Figure 5A, B). Interestingly, cell surface expression of CXCR4 was limited to a subset of TOX3^+^ (GFP^+^) cells (Figure 5B). Given the change in chemokine receptor expression, we asked whether TOX3 expression could influence cell migratory properties. We observed increased migration of cell lines expressing TOX3 in response to serum (Figure 5C, D).

Recently, it has been demonstrated that IGF-1 cooperates with CXCR4 signaling to promote bone marrow metastasis of breast cancer, potentially one factor in the aggressive behavior of LumB tumors [29]. Given that TOX3 upregulated CXCR4, enhanced the migratory properties of MCF-7 cells, and is highly expressed in LumB tumors, we asked whether IGF-1 could play a role in regulating TOX3 expression. Indeed, MCF-7 cells treated with IGF-1 showed significant, albeit modest, upregulation of *TOX3* expression (Figure 5E), as well as the known IGF-1 target gene *GAPDH* [30]. Together, these results offer a potential link between breast cancer metastasis and TOX3 expression.

### TOX3 regulates estrogen responsive genes in a ligand-independent manner

**Figure 6:**
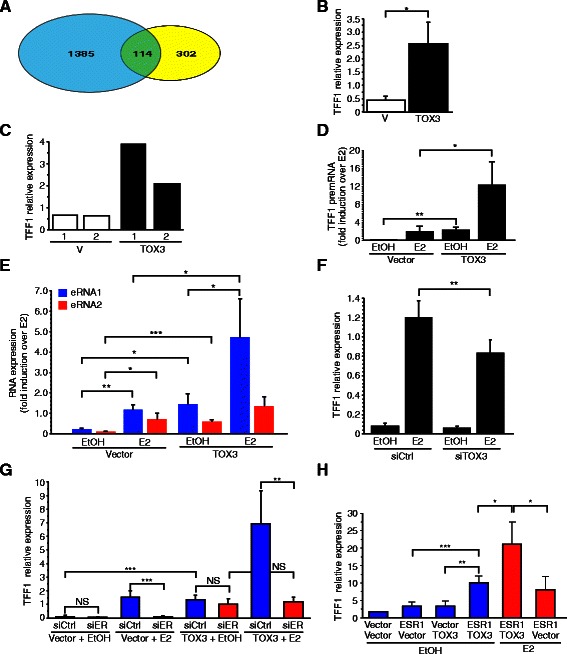
**TOX3 regulates estrogen-responsive genes in a ligand-independent manner. A**. Overlap of genes upregulated by TOX3 (yellow) in MCF-7 cells as shown here, and those previously reported to be bound by ER and/or be regulated by estrogen [31] (blue). **B**. *TFF1* mRNA expression in transiently transfected MCF-7 cells under estrogen depleted conditions. Data are expressed as mean ± SD of three independent experiments. **C**. *TFF1* gene expression in stably transfected MCF-7 cell lines. **D**. *TFF1* pre-mRNA expression in indicated cell lines treated with estrogen (E2) or vehicle (EtOH). **E**. eRNA expression in stably transfected MCF-7 cells. Data are expressed as mean ± SD of three independent experiments. **F**. Estrogen-induced *TFF1* mRNA expression following siRNA-mediated TOX3 knockdown as indicated. Data are expressed as mean ± SD of six independent experiments. **G**. *TFF1* expression following siRNA mediated ER knockdown in MCF-7 stable cells lines as indicated in presence of estrogen (E2) or vehicle (EtOH). Data are expressed as mean ± SD of four independent experiments. **H**. *TFF1* expression after ER and/or TOX3 transfection of MDA-MB-231 cells and with indicated additions. Data are expressed as mean ± SD of four independent experiments.

Approximately 27% of the genes whose expression was modulated by TOX3 in the above analysis have been previously reported to be regulated by estrogen in MCF-7 cells ([31] (Figure 6A). Given this, we were surprised that the well-characterized estrogen responsive gene *TFF1* [32,33] did not meet our stringent microarray threshold criteria. However, upon closer inspection this was due to variable basal expression of *TFF1* in the estrogen-starved cells used in the microarray experiment. Using qRT-PCR, however, we observed a significant and consistent upregulation of *TFF1* by TOX3 in estrogen depleted MCF-7 (Figure 6B). Consistent with the results from these transient transfections, two stably transfected independent MCF-7 cell lines that expressed TOX3 maintained higher *TFF1* expression in the absence of estrogen when compared to vector-transfected control MCF-7 cell lines (Figure 6C). To more accurately gauge whether *TFF1* was actively transcribed in the absence of estrogen, we analyzed *TFF1* pre-mRNA following 48 hours of estrogen depletion. As expected, *TFF1* pre-mRNA is induced by estrogen in vector transfected control cell lines (Figure 6D). In contrast, *TFF1* pre-mRNA is upregulated in TOX3 overexpressing cells compared to control cells even in the absence of estrogen (vehicle treated), and induced to very high levels in the presence of estrogen (Figure 6D). This suggests that the *TFF1* gene is not only upregulated by TOX3 in the absence of estrogen, but that TOX3 can enhance the estrogen responsiveness of this gene.

ER binding sites are found in the TFF1 promoter and in two upstream enhancer regions, and ER is thought to be involved in looping of the chromatin to juxtapose enhancers and promoter [34,35]. Recently, long non-coding enhancer RNAs (eRNA) have been shown to play a key role in the estrogen-mediated upregulation of *TFF1* in MCF-7 cells [36]. Treatment of MCF-7 cells with estrogen upregulated both of the reported *TFF1* eRNAs (transcribed from different strands) (Figure 6E). Interestingly, these same eRNAs were upregulated in TOX3 expressing MCF7 cells in the absence of estrogen (Figure 6E). Addition of estrogen to TOX3-expressing cells further upregulated eRNA1 (Figure 6E). A trend towards further upregulation of eRNA2 was seen in the presence of estrogen in TOX3-expressing cells, although this did not reach significance and was of lower magnitude than that seen for eRNA1. This may suggest that TOX3 can differentially regulate these two eRNAs.

Given these results, we asked whether TOX3 is involved in endogenous regulation of TFF1 expression in MCF-7 cells, despite low to undetectable expression of the protein in this cell line (Figure 4C). siRNA-mediated knockdown of TOX3 blunted the TFF1 response to estrogen treatment in MCF-7 cells (Figure 6F), suggesting that even low levels of TOX3 may impact estrogen activation of the *TFF1* gene.

As TOX3 can activate the *TFF1* gene in the absence of estrogen, we asked if ER was required for TOX3 regulation of *TFF1* gene expression. As expected, siRNA-mediated knockdown of ER inhibited estrogen mediated *TFF1* induction in MCF-7 (Figure 4G). Surprisingly, however, loss of ER did not affect *TFF1* expression in TOX3 stably expressing cells in the absence of estrogen (Figure 6G). In contrast, ER was required for estrogen upregulation of *TFF1* in TOX3-expressing cells (Figure 6G). Together, these data suggest that stable maintenance of *TFF1* expression in the presence of TOX3 and absence of estrogen is largely ER independent.

To address whether the induction of *TFF1* by TOX3 was ER-dependent, we transfected triple negative basal MDA-MB-231 breast cancer cells, which do not express ER or TOX3. Neither expression of TOX3 nor ER alone induced *TFF1* in MDA-MB-231 cells (Figure 6H). However, expression of both TOX3 and ER led to *TFF1* induction (Figure 6H). Moreover, cells expressing TOX3 and ER were more responsive to estrogen than cells expressing ER alone (Figure 6H).

## Discussion

We show here that murine *Tox3* is highly expressed in ER^+^ luminal progenitors and ER^+^ mature luminal cells, but not in ER^−^ luminal progenitors or basal epithelial cell populations. Taking advantage of the recently reported expression profiling of the human counterparts of these cells, we found a similar expression pattern in the human mammary gland.

The function of TOX3 in the mammary epithelium remains to be determined. However, that TOX3 has the potential to regulate a subset of ER target genes (see below) raises the possibility that TOX3 might play a similar role in normal mammary epithelium. Interestingly, ER^+^ progenitors have been shown to be resistant to *in vivo* estrogen deprivation [18]. Given our results of ligand-independent estrogen target gene upregulation by TOX3 in tumor cells, it is possible that TOX3 also contributes to the relative insensitivity of these progenitors to estrogen deprivation.

Two *TOX3* transcripts encoding proteins with distinct N-termini have been reported, and we found predominant expression of mRNA encoding the long form of TOX3 in normal breast tissue, breast cancer cell lines, and primary breast cancer. Using a rabbit anti-TOX3 monoclonal antibody specific for the long-form of the protein, we showed that TOX3 is exclusively a nuclear protein that is expressed in a subset of luminal epithelial cells, but not in myoepithelial cells or stroma, consistent with the expression pattern of the gene. Expression of TOX3 protein was not uniform, and areas exhibiting high levels of expression were seen in luminal epithelial cells in some samples. Areas enriched for TOX3-expressing cells were also highly enriched for epithelial cells expressing ER, PR, and FOXA1. Whether these TOX3^+^ islands represent ER^+^ progenitors and/or an ER^+^ subset of mature epithelial cells is not known. TOX3 expression was not associated with highly proliferative regions of the mammary gland as assessed by Ki67 staining (data not shown).

The coexpression of TOX3 and FOXA1 in a subset of luminal epithelial cells is interesting. The expression of TOX3 is regulated at least in part by FOXA1, an ER pioneer factor that is involved in delineating the luminal lineage [37-39]. FOXA1 is thought to be a positive regulator of *TOX3* expression, mediated by binding to an upstream enhancer [11], and knockdown of FOXA1 in a breast cancer cell line decreased *TOX3* expression [40]. We also observed modest *TOX3* upregulation in MCF-7 cells following IGF-1 treatment. This growth factor has been shown to contribute to breast cancer progression and endocrine resistance [29,41] through stabilization of FOXA1 protein in MCF-7 cells [42]. Moreover, FOXA1 alters the pattern of ER binding in ‘poor outcome/metastatic’ ER^+^ breast cancer from that found in ER^+^ ‘good outcome’ breast cancer [43]. Identification of genes within 20 kb of these differential ER binding sites led to a gene expression predictor set that included *TOX3*, with upregulation of *TOX3* associated with poor outcome patients. In normal cells, a FOXA1-TOX3 circuit may play a role during progenitor cell differentiation of the ER^+^ luminal cell subset. However, the disease risk allele SNP increases the affinity of FOXA1 for the *TOX3* upstream enhancer, inhibiting the function of this regulatory sequence and leading to a reduction in *TOX3* expression [11]. This suggests additional complexity in *TOX3* gene regulation, where a narrow range of FOXA1 binding may be key to appropriate enhancer function.

The *TOX3* gene was expressed across multiple molecular subtypes of breast cancers, and there was heterogeneity of expression within each subtype. The notable exception was basal tumors, which were generally *TOX3*^-/low^. The TOX3 protein was expressed in a significant proportion of histologically defined LumB tumors, and high expression of the *TOX3* gene in patients bearing LumB tumors was associated with poorer outcome. Expression profiling comparisons between LumB tumors and normal mammary epithelial cell population has suggested that these tumors are most similar to ER^+^ luminal progenitors and ER^+^ mature luminal cells [18,25], the two cell populations that normally express TOX3. Thus, ER^+^ luminal progenitors may be one origin of TOX3^+^ LumB tumors.

MCF-7 cells poorly express endogenous TOX3 protein. This is likely due to heterozygosity for the *TOX3* SNP affecting enhancer function [11]. Using MCF-7 cells, we found that TOX3 has the ability to acutely regulate key genes involved in cell cycle and metastases, two key features in breast cancer progression. Consistent with a role for TOX3 in proliferation of tumor cells, knockdown of TOX3 in BT474 inhibits growth in culture, while knockdown of TOX3 in ZR-75-1 led to poor tumor formation in nude mice [44].

We also found upregulation of CXCR4 and an increase in migration of TOX3-expressing MCF-7 cells. In conjunction with upregulation of *TFF1*, this may partly explain the finding that primary breast cancers that subsequently metastasize specifically to the bone are associated with upregulation of *TOX3* (*TNRC9* in [14]). Although TOX3 expression led to consistent upregulation of *CXCR4* at the cell population level, expression of this chemokine receptor was only evident on a subset of cells that expressed TOX3 (Figure 6B and data not shown). This may reflect the intrinsic stochastic nature of gene regulation [45,46], and raises the possibility that factors such as TOX3 that interact with DNA in a non-sequence specific fashion to modify chromatin [5], may alter the threshold of gene regulation and result in tumor cell heterogeneity that can then be acted upon by selective pressures.

The disease associated *TOX3* SNP has been shown to have an additive effect on disease risk in BRCA1 mutation carriers [47]. Recently, knockdown of TOX3 was reported to cause upregulation of BRCA1 in MCF-7 cells [44], thus implicating TOX3 as a BRCA1 repressor. In contrast, we observed upregulation of BRCA1 and BRCA2 (along with other genes related by network analysis) upon TOX3 expression, consistent with our observation that TOX3 primarily acts as a transcriptional activator. The reason for this apparent discrepancy is unclear, although our experiments were carried out under estrogen-depleted conditions, which might alter BRCA1 regulation.

We also noted significant overlap between genes whose expression was altered by TOX3 and genes regulated by estrogen in MCF-7 cells [31]. This may reflect the direct action of TOX3 on estrogen responsive elements [17]. Given that we performed our microarray analysis under estrogen-depleted conditions, this suggests that TOX3 can regulate a subset of estrogen responsive genes in a ligand-independent manner. Indeed, this was demonstrated for the well-characterized ER target gene *TFF1*, which was induced by TOX3 under estrogen-depleted conditions and in MDA-MB-231 following co-transfection of TOX3 and ER. While the exact mechanism of action of TOX3 remains to be elucidated, TOX3 was able to induce *TFF1* eRNAs even in the absence of estrogen, possibly indicative of promotion of a similar looping mechanism to that of ER and its ligand. Moreover, although we could not extinguish *TFF1* expression in TOX3-expressing MCF7 by inhibition of ER, ER and TOX3 together were sufficient to induce TFF1 expression in MDA-MB-231 cells in the absence of estrogen. Thus, the relationship of ER and TOX3 in the absence of estrogen may be complex, as these data suggest that TOX3-mediated induction but not maintenance of *TFF1* gene expression is ER-dependent. Alternatively, other cofactors that differ between MCF7 and MDA-MB-231 cells may complicate the interpretation of these results. Nevertheless, unliganded ER has recently been shown to play a significant role in gene regulation in breast cancer cells [48], and we would propose that TOX3 plays a modulatory role on this activity.

Additionally, we observed hyper-responsive *TFF1* gene expression following estrogen treatment in cells overexpressing TOX3. Expression of TOX3 may be involved in the resistance to endocrine therapy reported to occur in some LumB cancers [41]. Consistent with this, cells overexpressing TOX3 were better able to survive under estrogen-deprived conditions.

## Conclusions

Together, our data suggest that TOX3 expression within the context of breast cancer is likely a tumor promoter rather than a strict tumor suppressor as had been proposed based on the decrease in *TOX3* expression associated with disease risk [11]. In addition, TOX3 has the potential to regulate ER target gene expression in the face of limiting concentrations of estrogen, of potential importance in considering therapeutic modalities for TOX3^+^ breast cancer. One key issue to be resolved is how a decrease in TOX3 expression alters the mammary gland. BRCA1 mutation carriers have an accumulation of ER^−^ luminal progenitors, the presumed cell of origin for triple negative tumors [49-52]. One possibility is that individuals carrying the TOX3 disease-risk allele similarly have an accumulation of a cell population that is more susceptible to tumorigenesis. Given our findings that TOX3 is expressed in luminal progenitors and that family member TOX regulates cell differentiation [7,8,53], lower TOX3 expression may alter the equilibrium of luminal cell populations in favor of a progenitor state that is more susceptible to tumorigenesis. This might also explain the association of the *TOX3* risk allele with triple negative breast cancer [54,55]. However, once cell transformation is initiated, TOX3 likely plays a significant role in rendering resulting tumors more aggressive and metastatic, and thus may serve as a novel biomarker for LumB breast cancer.

## Additional files

Additional file 1: Figure S1. TOX3 expression in human mammary epithelial cell populations. Previously published gene expression data of indicated cell populations were mined for *TOX3* expression. Data are expressed as mean ± SD of normalized hybridization signals reported by Shehata et al. [18].
Additional file 2: Figure S2. Validation of anit-TOX3 rabbit monoclonal antibody AJ-33. A. Shown is a Western blot probed with AJ-33 along with qRT-PCR analysis of mixes of TOX3-transfected and non-transfected HEK293T cells, as indicated. B. Immunofluorescence of HEK293T cells transfected with either vector or TOX3 expression plasmid, stained with AJ-33 antibody and counterstained with DAPI. Both vectors also express IRES-regulated GFP. Arrows indicate examples of transfected (GFP^+^) cells.
Additional file 3: Figure S3. Expanded heat map of Figure 4 containing gene names.
Additional file 4: Figure S4. Ingenuity pathway analysis of gene expression changes, including pathways involved in cell cycle and DNA repair.
