# Supplementary figures and images for: TOX3 is expressed in mammary ER+ epithelial cells and regulates ER target genes in luminal breast cancer
Source: BMC Cancer. 2015 Jan 30;15:22. doi: 10.1186/s12885-015-1018-2 (PMC4324787; doi:10.1186/s12885-015-1018-2)

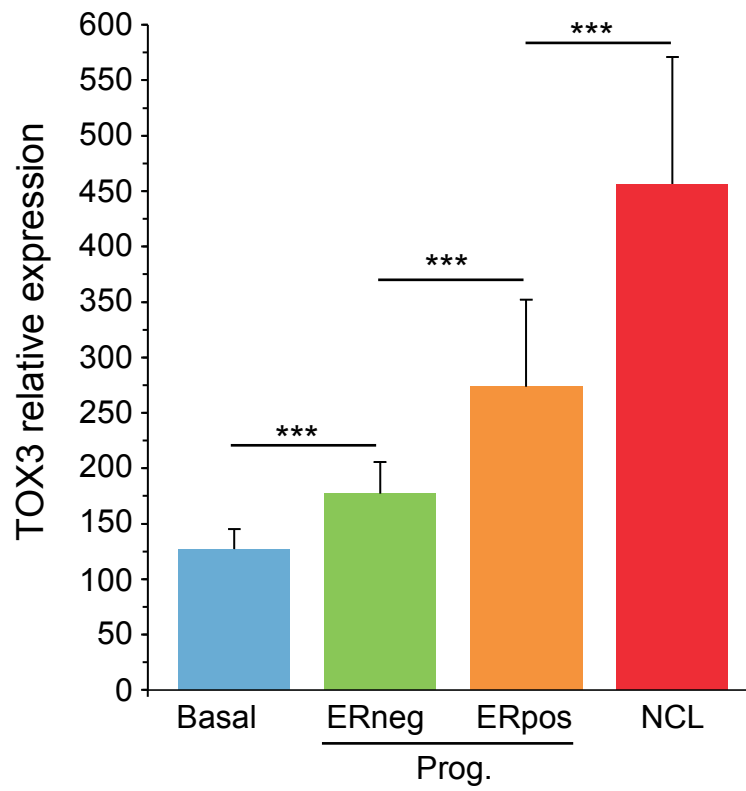

Supplement: Additional file 1: Figure S1. — TOX3 expression in human mammary epithelial cell populations. Previously published gene expression data of indicated cell populations were mined for TOX3 expression. Data are expressed as mean ± SD of normalized hybridization signals reported by Shehata et al. [18]. [file 12885_2015_1018_MOESM1_ESM.pdf]

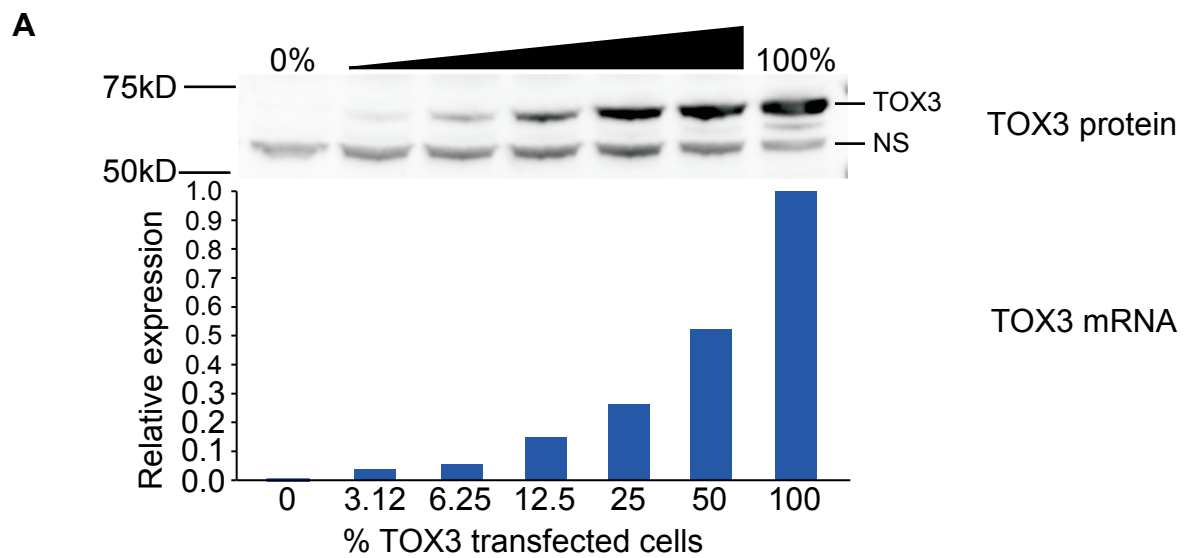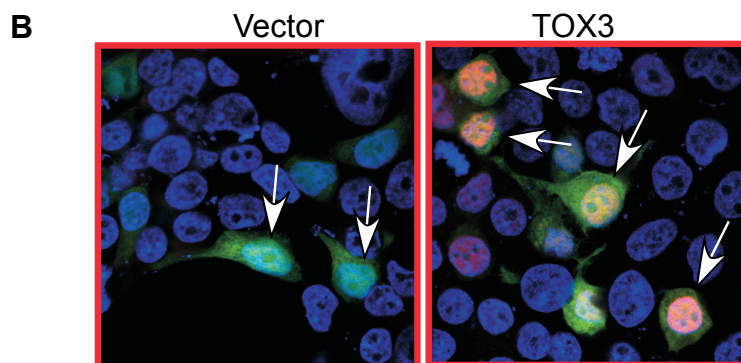

Supplement: Additional file 2: Figure S2. — Validation of anit-TOX3 rabbit monoclonal antibody AJ-33. A. Shown is a Western blot probed with AJ-33 along with qRT-PCR analysis of mixes of TOX3-transfected and non-transfected HEK293T cells, as indicated. B. Immunofluorescence of HEK293T cells transfected with either vector or TOX3 expression plasmid, stained with AJ-33 antibody and counterstained with DAPI. Both vectors also express IRES-regulated GFP. Arrows indicate examples of transfected (GFP+) cells. [file 12885_2015_1018_MOESM2_ESM.pdf]

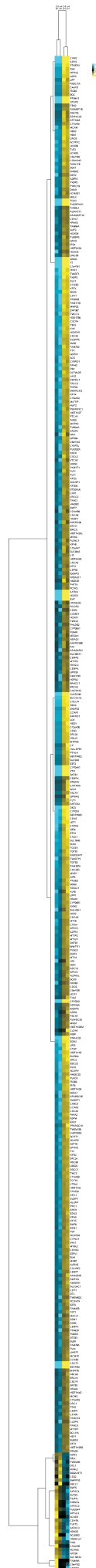

Supplement: Additional file 3: Figure S3. — Expanded heat map of Figure 4 containing gene names. [file 12885_2015_1018_MOESM3_ESM.pdf]

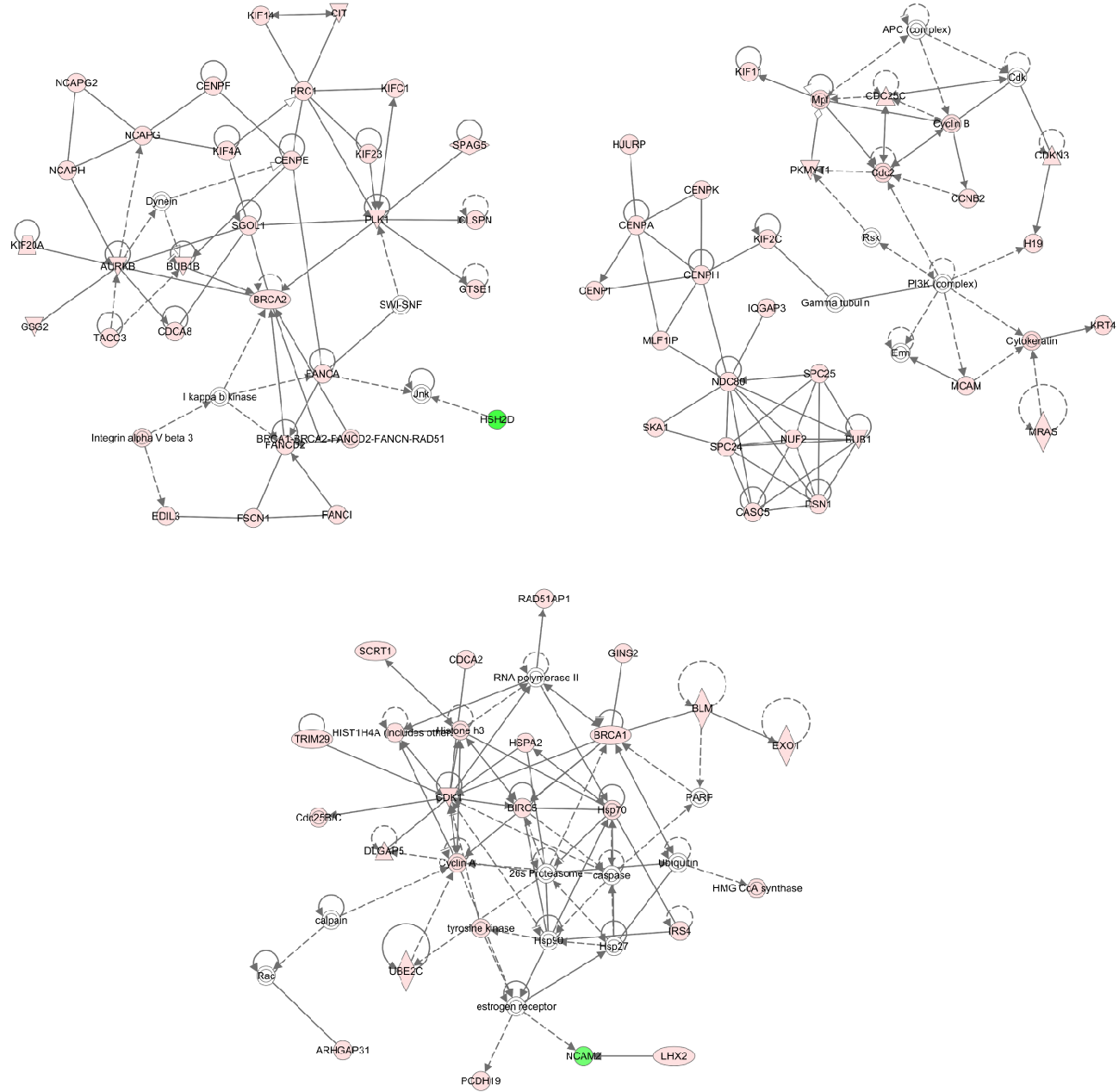

Supplement: Additional file 4: Figure S4. — Ingenuity pathway analysis of gene expression changes, including pathways involved in cell cycle and DNA repair. [file 12885_2015_1018_MOESM4_ESM.pdf]
